# Letermovir Prophylaxis for Cytomegalovirus Infection in Allogeneic Stem Cell Transplantation: A Real-World Experience

**DOI:** 10.3389/fonc.2021.740079

**Published:** 2021-09-06

**Authors:** Massimo Martino, Annalisa Pitino, Mercedes Gori, Benedetto Bruno, Alessandra Crescimanno, Vincenzo Federico, Alessandra Picardi, Stefania Tringali, Claudia Ingrosso, Paola Carluccio, Domenico Pastore, Gerardo Musuraca, Annalisa Paviglianiti, Adriana Vacca, Bianca Serio, Gabriella Storti, Nicola Mordini, Salvatore Leotta, Michele Cimminiello, Lucia Prezioso, Barbara Loteta, Anna Ferreri, Fabrizia Colasante, Emanuela Merla, Luisa Giaccone, Alessandro Busca, Maurizio Musso, Renato Scalone, Nicola Di Renzo, Serena Marotta, Patrizio Mazza, Pellegrino Musto, Immacolata Attolico, Carmine Selleri, Filippo Antonio Canale, Marta Pugliese, Giovanni Tripepi, Gaetana Porto, Giovanni Martinelli, Angelo Michele Carella, Claudio Cerchione

**Affiliations:** ^1^Centro Unico Regionale Trapianti Cellule Staminali e Terapie Cellulari (CTMO), Grande Ospedale Metropolitano “Bianchi-Melacrino-Morelli”, Reggio Calabria, Italy; ^2^Istituto di Fisiologia Clinica del Consiglio Nazionale delle Ricerche (CNR), Roma, Italy; ^3^Dipartimento di Oncologia, SSD Trapianto Allogenico di Cellule Staminali, AOU Città della Salute e della Scienza di Torino, Torino, Italy; ^4^Dipartimento di Biotecnologie Molecolari e Scienze per la Salute, Divisione di Ematologia, Università di Torino, Torino, Italy; ^5^Unità Operativa di Oncoematologia e TMO, Istituto “La Maddalena”, Palermo, Italy; ^6^Ematologia e Trapianto di Cellule Staminali, Polo Ospedaliero “Vito Fazzi”, Lecce, Italy; ^7^UOC Ematologia con Trapianto CSE, AORN “Antonio Cardarelli”, Napoli, Italy; ^8^Dipartimento di Biomedicina e Prevenzione, Università di Roma Tor Vergata, Roma, Italy; ^9^UOSD UTMO, AOR Villa “Sofia Cervello”, Palermo, Italy; ^10^Ematologia e Trapianto di Midollo Osseo, Ospedale “San Giuseppe Moscati”, Taranto, Italy; ^11^UOC di Ematologia con Trapianto, Dipartimento di Emergenza e Trapianti d’Organo, Università degli Studi “Aldo Moro” e AOUC Policlinico di Bari, Bari, Italy; ^12^Divisione di Ematologia, Ospedale “Antonio Perrino”, Brindisi, Italy; ^13^Unità di Ematologia, IRCCS Istituto Romagnolo per lo Studio dei Tumori (IRST) “Dino Amadori”, Meldola, Italy; ^14^UO Ematologia - CTMO, Polo Ospedaliero “Armando Businco”, Cagliari, Italy; ^15^Dipartimento di Medicina, Chirurgia e Odontoiatria, Università di Salerno, Salerno, Italy; ^16^Unità di Ematologia, Azienda Ospedaliera “San Giuseppe Moscati”, Avellino, Italy; ^17^SC Ematologia, Azienda Ospedaliera “S. Croce e Carle”, Cuneo, Italy; ^18^Programma di Trapianto Emopoietico, Azienda Policlinico “Vittorio Emanuele”, Catania, Italy; ^19^Divisione Universitaria di Ematologia, Ospedale “San Carlo”, Potenza, Italy; ^20^Ematologia e Centro Trapianti Midollo Osseo (CTMO), Dipartimento ad Attività Integrata Medicina Generale e Specialistica, Azienda Ospedaliero-Universitaria di Parma, Parma, Italy; ^21^Ospedale I.R.C.C.S. Casa Sollievo della Sofferenza - Centro Trapianti di Cellule Staminali, San Giovanni Rotondo, Italy; ^22^Istituto di Fisiologia Clinica del Consiglio Nazionale delle Ricerche (CNR), Reggio Calabria, Italy

**Keywords:** cytomegalovirus infection, allogeneic stem cell transplantation, prophylaxis, real-world data, Letermovir

## Abstract

Despite effective treatments, cytomegalovirus (CMV) continues to have a significant impact on morbidity and mortality in allogeneic stem cell transplant (allo-SCT) recipients. This multicenter, retrospective, cohort study aimed to evaluate the reproducibility of the safety and efficacy of commercially available letermovir for CMV prophylaxis in a real-world setting. Endpoints were rates of clinically significant CMV infection (CSCI), defined as CMV disease or CMV viremia reactivation within day +100-+168. 204 adult CMV-seropositive allo-SCT recipients from 17 Italian centres (median age 52 years) were treated with LET 240 mg/day between day 0 and day +28. Overall, 28.9% of patients underwent a haploidentical, 32.4% a matched related, and 27.5% a matched unrelated donor (MUD) transplant. 65.7% were considered at high risk of CSCI and 65.2% had a CMV seropositive donor. Low to mild severe adverse events were observed in 40.7% of patients during treatment [gastrointestinal toxicity (36.3%) and skin rash (10.3%)]. Cumulative incidence of CSCI at day +100 and day +168 was 5.4% and 18.1%, respectively, whereas the Kaplan-Meier event rate was 5.8% (95% CI: 2.4-9.1) and 23.3% (95% CI: 16.3-29.7), respectively. Overall mortality was 6.4% at day +100 and 7.3% at day +168. This real-world experience confirms the efficacy and safety of CMV.

## Introduction

Clinically significant cytomegalovirus (CMV) infection (CSCI), defined as CMV disease or CMV viremia reactivation after allogeneic stem-cell transplantation (allo-SCT), is often a serious complication given the delayed immune recovery of the host (1–3). Post-transplant CSCI varies from 30% to 70% and has been associated with higher non-relapse mortality (NRM) (4–10). During the past few decades, both clinical trials and real-world experiences have evaluated the role of CMV prophylaxis, reporting conflicting results (11, 12).

Letermovir (LET) is an antiviral agent with a novel mechanism of action characterized by inhibition of the CMV DNA terminase complex (13, 14). In a pivotal registration Phase 3 clinical trial, prophylaxis with LET significantly reduced the incidence of CSCI after allo-SCT (15). The drug was granted fast-track status by the US Food and Drug Administration (FDA), and orphan drug status by the European Medicines Agency. In the US and Europe, LET was approved for prophylaxis of CSCI in adult CMV-seropositive recipients of allo-SCT (16). FDA considers it a first-in-class medication (17).

The majority of Italian transplant programs adopted prophylaxis with LET as standard policy as soon as the drug became commercially available. The aims of the present multicenter, retrospective, cohort study were to investigate whether the results reported in the aforementioned phase 3 trial could be reproduced in a real-world experience and to assess whether prophylaxis could affect pre-emptive CMV therapy.

## Methods

Seventeen Italian Transplant Centers took part in the study. The observation period began in January 2019 when the Italian Medicines Agency (AIFA) authorized LET for commercial purposes. Prophylaxis was indicated by AIFA for patients aged 18 years or older who had positive CMV serologic status with an undetectable level of CMV DNAemia in whole blood before transplant. Patients received LET 480 mg tablet once daily between day 0 and day +28 after allo-SCT and continued until day +100 if no adverse events occurred during the observation period. If LET was co-administered with cyclosporine, the dosage of LET has been decreased to 240 mg once daily. The intravenous (IV) formulation of LET was not available in Italy. All patients continued herpesvirus prophylaxis as per standard practice.

A high sensitivity and high specificity serologic test was used to detect CMV-IgG before transplant and real-time PCR assay was used for post allo-SCT CMV monitoring (18). DNAemia was determined at least once a week in the first three months, and every other week in the second three-month period. Pre-emptive treatment was initiated when the CMV-DNAemia level was >1,000 copies/mL in plasma or 10,000 copies/mL in whole blood, in two consecutive assessments (18, 19). Patients were evaluated up to day +100 for the primary efficacy endpoint, after which follow-up continued through week 24 post-transplant.

Conditioning intensity was classified according to Working Group definitions (20). Donor selection, conditioning, graft-versus-host disease (GVHD) prophylaxis, and supportive care followed standard institutional operating procedures (21).

Patients were considered at high risk of CSC if one or more of the following criteria were present: human leukocyte antigen (HLA)-related (sibling) donor with at least one mismatch at HLA-A, -B or -DR loci; haploidentical donor; unrelated donor with at least one mismatch at HLA-A, -B, -C or -DRB; use of umbilical cord blood as stem cell source; use of *ex vivo* T-cell-depleted grafts; ≥grade 2 GVHD requiring systemic corticosteroids. Given the retrospective design (i.e., the non-interventional nature) of the study, no sample size calculation was performed.

### Endpoints

The primary endpoint was the incidence of CSCI leading to pre-emptive treatment (22) at day +100 (14 weeks) post allo-SCT, and the time to CSCI. The secondary endpoint was the incidence of CSCI at day +168 (24 weeks). Follow-up time was calculated from the transplant date to the first positive CMV DNAemia or its last measurement during the study period, whichever occurred first. Censoring time was the last date of the positive CMV test or the date of death if <14 days from the last negative test. Since endpoints were evaluated at day +100 and day +168, no data on CMV tests were collected after day +168.

### Statistical Analysis

The follow-up period (spanning from January 2019 to June 2020) was calculated as the time (in days) spanning from the transplant date to the first positive CMV DNAemia (*i.e.*, the achievement of the study endpoint) or the last observation coinciding with 168 days or the date of death or lost to follow-up. Data were summarized as median and interquartile range, or absolute number and percentage. To identify the demographic and clinical correlates of CMV infection and drug discontinuation we did not use a face-to-face comparison of patients’ characteristics but the univariate logistic analysis, a method that specifically allows to assess the strength of the risk factor-study outcomes links. Bonferroni’s correction was used to minimize the possibility of false-positive findings due to multiple testing. Kaplan-Meier survival analysis was performed for time to infection, overall-survival (OS), and non-relapse mortality (NRM). NRM was defined as death without recurrent or progressive disease after allo-SCT. Probabilities of NRM were estimated with the use of cumulative incidence curves, with relapse viewed as a competing risk. Gray’s method was used to evaluate the differences between groups (23). If no competitive risk was found, a standard Kaplan-Meier analysis was applied. Data were analysed with STATA/IC 13.1 for Windows (College Station, TX) and RStudio-1.2.5033.1.

### Compliance With Ethical Standards

This multicenter, retrospective, cohort study was approved by the Ethics Committee of the coordinating centre, Grande OspedaleMetropolitano “Bianchi-Melacrino-Morelli” of Reggio Calabria, Italy, and by those of the other participating centres. All procedures were performed according to the principles laid down in the 1964 Helsinki declaration and its later amendments or comparable ethical standards. All patients signed the informed consent.

## Results

Overall, 230 patients who underwent an allograft in the participating Italian centres were enrolled; 26 were excluded from the analysis because of incomplete data. Median age of the 204 patients in the study cohort was 52 years, and 53.9% were males (**Table 1**). Acute myeloid leukaemia (AML) was diagnosed in 53.4%. Overall, 28.9% underwent a haploidentical, 32.4% a matched related (MRD), and 27.5% a matched unrelated donor (MUD) transplant. In the majority of patients, stem cell source was G-CSF mobilized peripheral blood (65.7%). 65.7% of patients were considered at high risk of CMV and 65.2% had a CMV seropositive donor. The first patient was enrolled in January 2019 and the last one in June 2020.

**Table 1:** Baseline patients’ characteristics.

Median age, years (range)	52 (18-75)
Male gender n. (%)	110 (53.9)
CMV-seropositive donor n. (%)	133 (65.2)
**Disease** n. (%)	
Acute myeloid leukaemia	109 (53.4)
Myelodysplastic syndrome	19 (9.3)
Non-Hodgkin’s lymphoma	15 (7.4)
Acute lymphocytic leukaemia	28 (13.7)
Other disease	33 (16.2)
**HLA matching and donor type** n. (%)	
Matched unrelated	56 (27.5)
Matched related	66 (32.4)
Mismatched related	68 (33.3)
Mismatched unrelated	14 (6.9)
Haploidentical-related donor n. (%)	59 (28.9)
**Stem-cell source** n. (%)	
Peripheral blood	175 (85.8)
Bone marrow	27 (13.2)
Cord blood	2 (1)
Myeloablative conditioning regimen n. (%)	134 (65.7)
Acute GVHD grade ≥2 on the day of starting letermovir n. (%)	4 (2)
**Risk of CMV disease** n. (%)	
High risk	134 (65.7)
Low risk	70 (34.3)
ATG n. (%)	98 (48.0)
*Ex vivo* T-celldepletion n. (%)	16 (7.8)
Cyclosporine n. (%)	66 (32.4)
Tacrolimus n. (%)	135 (66.2)
Mycophenolate n. (%)	194 (95.1)

CMV, cytomegalovirus; HLA, human leukocyte antigen; GVHD, graft-versus-host disease, ATG, anti-thymocyte globulin.

### Incidence of CMV Infection and Discontinuation

**Figure 1:**
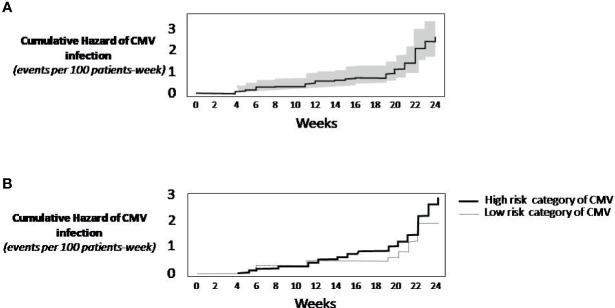
Kaplan-Meier Survival Analysis. **(A)** Cumulative rate of CMV infection (continuous line). The grey area around the continuous line represents the 95% confidence intervals. **(B)** Cumulative incidence of CMV infection by risk category of CMV. The analysis did not consider the competitive risk of mortality. When not considering the competing mortality risk, the practical implication is that the analysis of CMV infection censors patients who die. As this censoring is assumed to be uninformative, the resulting prognosis should be interpreted as the risk of CMV infection in a hypothetical setting in which patients do not die.

The cumulative incidence of CSCI was 5.4% at day +100 and 18.1% at day +168 after transplant (**Table 2**). Overall, from day +100 to day +168, the cumulative incidence of CSCI was 12.7%. Twenty patients discontinued the trial in the first 100 days, the majority (13 patients) because of death. The percentage of discontinuation was 28.4% at 168 days. Thirteen (6.4%) patients died before day +100 day and 15 (7.3%) before day +168. The Kaplan-Meier event rate of CSCI through 24 weeks was 23.3% (95% CI: 16.3-29.7), 5.8% at day +100 (95% CI: 2.4-9.1). Of note, starting at week +19, the incidence of CMV infection rapidly increased (**Figure 1A**). Given that no competitive risk from drug discontinuation was found on incidence rate by baseline CMV risk categories, low versus high, (competitive risk: 14 weeks, *p*=0.15; 24 weeks, *p*=0.84), a standard Kaplan-Meier analysis showed substantially similar curves (**Figure 1B**).

**Table 2:** Cumulative incidence of CMV infection and discontinuation at 100 days and at 168 days in 204 patients.

	No.	% (95% CI)
Clinically significant CMV infection through week 14 after transplantation	11	5.4 (2.3-8.5)
Initiation of pre-emptive therapy	4	2.0 (0.1-3.9)
CMV disease	5	2.5 (0.3-4.6)
Discontinued before 100 days without CMV	20	9.8 (5.7-13.9)
Patients who died	13	6.4 (3.0-9.7)
Clinically significant CMV infection through week 24 after transplantation	37	18.1 (12.8-23.4)
Initiation of pre-emptive therapy	17	8.3 (4.5-12.1)
CMV disease	7	3.4 (0.9-5.9)
Discontinued before 168 days without CMV	58	28.4 (22.2-34.6)
Patients who died	15	7.3 (3.8-10.9)

CMV, cytomegalovirus; 95% CI 95%, confidence interval.

### Adverse Events

Over 100 days of treatment, 40.7% of patients treated with LET experienced adverse events (AEs) (**Table 3**). Gastrointestinal AEs were the most frequent (36.3%), followed by skin rash (10.3%). Six cases were CMV-positive; four were CMV negative and died before 100 days. LET was discontinued in 20 patients before 100 days.

**Table 3:** Adverse events to Letermovir. The frequency is given in percentage.

Adverse events	Frequency
Gastrointestinal	36.3
Rash	10.3
Cough	1.0
Peripheral edema	2.0
Fatigue	4.4
Head pain	2.9
Acute renal damage	3.4
Hypertension	3.9

### Correlates of Time to Drug Discontinuation and CMV Infection

After Bonferroni’s correction, no baseline characteristics were significantly associated with time to drug discontinuation or CMV infection at day +100 and at day +168 (**Tables 4** and **5**).

**Table 4:** Univariate logistic regression analysis of treatment discontinuation through first 100 days and through 168 days by baseline characteristics.

		14 weeks				24 weeks			
		No discontinuation	Discontinuation	OR (95% CI)	*p*	No discontinuation	Discontinuation	OR (95% CI)	*p*
Age, years	≤52	97	7	1		79	25	1	
	>52	87	13	2.07 (0.79-5.42)	0.2	67	33	1.55 (0.84-2.87)	0.2
Gender	Female	87	7	1		72	22	1	
	Male	97	13	1.67 (0.64-4.36)	0.3	74	36	1.59 (0.86-2.96)	0.1
CMV donor	Negative	63	8	1		52	19	1	
	Positive	121	12	0.78 (0.30-2.01)	0.6	94	39	1.14 (0.60-2.16)	0.7
Disease*	Acute myeloid leukaemia	99	10	1		80	29	1	
	Myelodysplastic syndrome	19		0.5 (0.11-1.72)	0.3	14	5	0.99 (0.3-2.83)	0.9
	Non-Hodgkin’s lymphoma	14	1			9	6	1.84 (0.57-5.56)	0.3
	Acute lymphocytic leukaemia	26	2			23	5	0.6 (0.19-1.62)	0.3
	Other disease	26	7	2.67 (0.89-7.64)	0.07	20	13	1.79 (0.78-4.04)	0.2
HLA	Matched unrelated	47	9	1		37	19	1	
	Matched related	63	3	0.25 (0.05-0.88)	0.04***	50	16	0.62 (0.28-1.37)	0.2
	Mismatched related	63	5	0.41 (0.12-1.28)	0.14	50	18	0.7 (0.32-1.52)	0.4
	Mismatched unrelated	11	3	1.42 (0.28-5.75)	0.64	9	5	1.08 (0.3-3.6)	0.9
Haploidentical-related donor	No	129	16	1		104	41	1	
	Yes	55	4	0.59 (0.19-1.83)	0.4	42	17	1.03 (0.52-1.98)	0.9
Stem source	Peripheral blood	156	19	1		124	51	1	
	Other source	28	1	0.29 (0.04-2.28)	0.2	22	7	0.77 (0.29-1.84)	0.6
Myeloablative regimen	No	60	10	1		43	27	1	
	Yes	124	10	0.48 (0.19-1.22)	0.1	103	31	0.48 (0.26-0.9)	0.02***
ATG	No	99	7	1		79	27	1	
	Yes	85	13	2.16 (0.82-5.67)	0.1	67	31	1.35 (0.74-2.5)	0.3
*Ex vivo* cell depletion**	No	168	20			132	56	1	
	Yes	16			–	14	2	0.34 (0.05-1.26)	0.1
Cyclosporine	No	125	13	1		98	40	1	
	Yes	59	7	1.14 (0.43-3.01)	0.8	48	18	0.92 (0.48-1.77)	0.8
Tacrolimus	No	62	7	1		51	18	1	
	Yes	122	13	0.94 (0.36-2.49)	0.4	95	40	1.19 (0.62-2.29)	0.6
Mycophenolate	No	8	2	1		8	2	1	
	Yes	176	18	0.41 (0.8-2.07)	0.3	138	56	1.62 (0.33-7.88)	0.5
Risk of CMV disease	Low	66	4	1		49	21	1	
	High	118	16	2.24 (0.72-6.97)	0.2	97	37	0.89 (0.47-1.7)	0.7

CMV, cytomegalovirus; HLA, human leukocyte antigen; ATG, anti-thymocyte globulin; No disc, not discontinued; Disc, discontinued; OR (95% CI) odds radio (95% confidence interval).

*For analysis at 14 weeks, disease was grouped in 3 classes; acute myeloid leukaemia; myelodysplastic syndrome - non-Hodgkin’s lymphoma - acute lymphocytic leukaemia; and other.

**For analysis at 14 weeks, no OR was calculated for ex vivo cell depletion.

***Not significant after Bonferroni’s correction.

**Table 5:** Univariate logistic regression analysis of CMV infection through first 100 days and through 168 days by baseline characteristics.

	Risk categories	14 weeks				24 weeks			
		CMV-	CMV+	OR (95% CI)	*P*	CMV-	CMV+	OR (95% CI)	*P*
Age, years	≤52	99	5	1		88	16	1	
	>52	94	6	1.26 (0.37-4.28)	0.9	79	21	1.46 (0.71-3.00)	0.4
Gender	Female	90	4	1		81	13	1	
	Male	103	7	1.53 (0.43-5.39)	0.5	86	24	1.74 (0.83-3.64)	0.1
CMV donor	Negative	67	4	1		54	17	1	
	Positive	126	7	0.93 (0.26-3.29)	0.9	113	20	0.56 (0.27-1.16)	0.1
Disease*	Acute myeloid leukaemia	100	9	1		94	15	1	
	Myelodysplastic syndrome	19		0.24 (0.04-0.96)	0.07	12	7	3.66 (1.2-10.69)	0.02****
	Non-Hodgkin’s lymphoma	15				14	1	0.45 (0.02-2.49)	0.5
	Acute lymphocytic leukaemia	26	2			22	6	1.71 (0.56-4.75)	0.3
	Other disease	33				25	8	2.01 (0.74-5.18)	0.2
HLA**	Matched unrelated	53	3	1		48	8	1	
	Matched related	63	3	0.84 (0.15-4.71)	0.8	56	10	1.07 (0.39-3.01)	0.9
	Mismatched related	63	5	1.15 (0.27-5.78)	0.9	55	13	1.42 (0.55-3.86)	0.5
	Mismatched unrelated	14				8	6	4.5 (1.21-16.82)	0.02****
Haploidentical-related donor	No	139	6	1		120	25	1	
	Yes	54	5	2.15 (0.63-7.32)	0.2	47	12	1.23 (0.57-2.63)	0.6
Stem source	Peripheral blood	165	10	1		142	33	1	
	Other source	28	1	0.59 (0.07-4.78)	0.6	25	4	0.69 (0.22-2.11)	0.5
Myeloablative regimen	No	66	4	1		57	13	1	
	Yes	127	7	0.91 (0.26-3.22)	0.9	110	24	0.96 (0.45-2.02)	0.9
Antimycotic regimen	No	101	5	1		86	20	1	
	Yes	92	6	1.32 (0.39-4.46)	0.7	81	17	0.9 (0.44-1.84)	0.8
Ex vivo***	No	177	11		—	154	34	1	
	Yes	16				13	3	1.05 (0.28-3.87)	0.9
Cyclosporine	No	132	6	1		115	23	1	
	Yes	61	5	1.8 (0.53-6.14)	0.3	52	14	1.35 (0.64-2.82)	0.4
Tacrolimus	No	64	5	1		55	14	1	
	Yes	129	6	0.6 (0.17-2.02)	0.4	112	23	0.81 (0.38-1.69)	0.6
Mycophenolate	No	9	1	1		7	3	1	
	Yes	184	10	0.49 (0.06-4.25)	0.5	160	34	0.5 (0.12-2.01)	0.3
**Risk of CMV disease**	Low	67	3	1		60	10	1	
	High	126	8	1.42 (0.36-5.52)	0.5	107	27	1.51 (0.69-3.34)	0.3

CMV, cytomegalovirus; CMV-, no CMV infection; CMV+, CMV infection; HLA, human leukocyte antigen; ATG, anti-thymocyte globulin; OR (95% CI), odds radio (95% confidence interval).

*For analysis at 14 weeks, disease was grouped into 2 classes; acute myeloid leukaemia and all others.

**For analysis at 14 weeks, HLA-matching donors were grouped into 3 classes; matched unrelated, matched related and mismatched.

***For analysis at 14 weeks, no OR was calculated for ex vivo cell depletion.

****Not significant after Bonferroni’s correction.

### GVHD

Incidences of acute GvHD grades II-IV by 168 days was 2% (4 patients) (**Table 1**). No patients experienced chronic GvHD during the period of follow-up

### Survival

**Figure 2:**
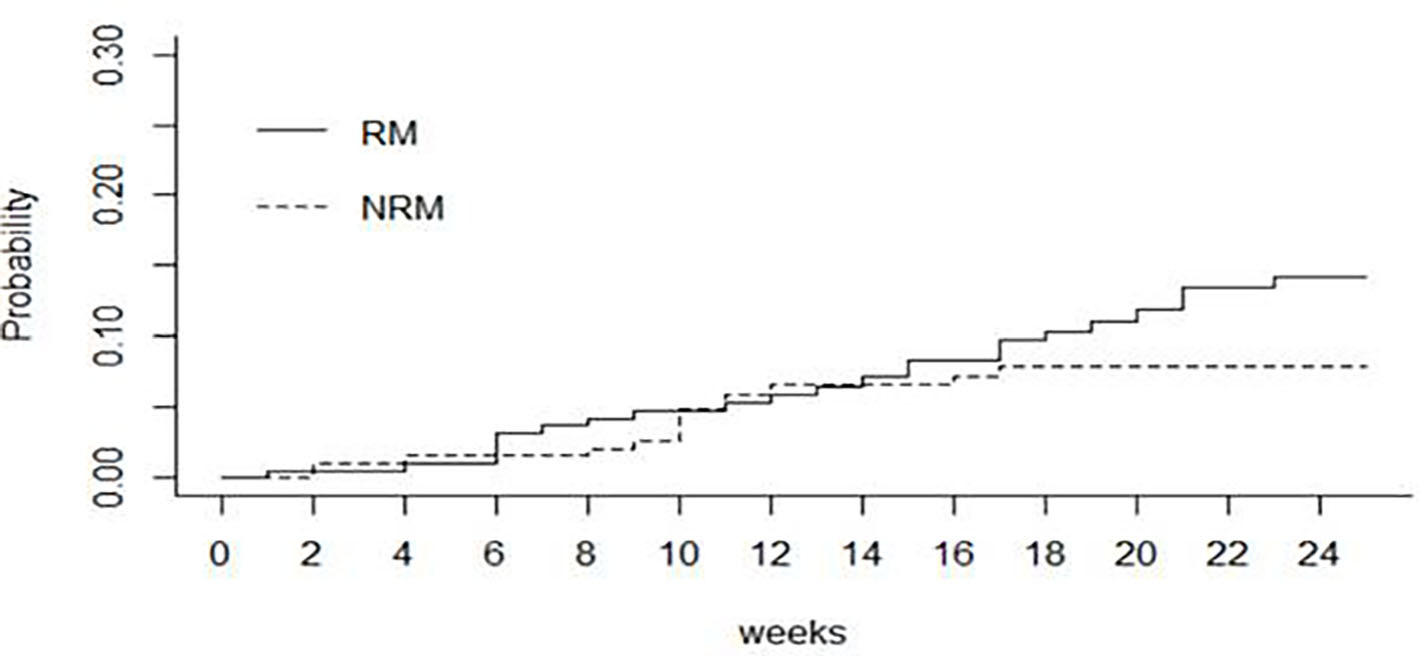
Cumulative incidence by relapse (RM) and non-relapse mortality (NRM).

4 patients with incomplete data set for OS and EFS have been excluded from the analysis; 200 patients were eligible for survival analysis. The cumulative incidence of death was 9.9% (95% CI 5.4-14.1) at day +100 and 14.3% (95% CI 8.7-19.5) at day +168 after transplant, respectively. Among patients who died (n=24 patients),10 had relapse. **Figure 2** shows the cumulative incidence of relapse mortality (RM) and non-relapse mortality (NRM), taking into account the competing risk of relapse. Overall, the cumulative incidences of NRM through the first 14 and 24 weeks were 7% (95% CI 4.0-11.0) and 8% (95% CI 4.0-12.0) respectively. The cumulative incidences of RM were 7% (95% CI 4.0-12.0) through the first 14 weeks and 14% (95% CI 9.0-20.0) through 24 weeks.

## Discussion

This multicenter, retrospective, cohort study shows that the cumulative incidence of CSCI was 5.4% and 18.1% at day +100 and at day +168 after transplant, respectively. Twenty patients discontinued the trial in the first 100 days, the majority (13 patients) because of death.

CSCI has been associated with increased NRM in transplant patients (2–6). Up until the introduction of LET, no antiviral prophylaxis had proven capable of preventing CSCI in seropositive patients. In randomized studies, prophylaxis with IV ganciclovir reduced the risk of CSCI without improving survival, while high doses of aciclovir or valaciclovir reduced the risk of CMV viremia reactivation but not of CMV disease (24–28). No differences in the risk of CMV disease or in patient survival were observed between prophylaxis with ganciclovir and valaciclovir (29), or between ganciclovir prophylaxis and pre-emptive therapy (9). Prophylaxis with foscarnet has only been used in uncontrolled trials, and its prolonged use is commonly limited by toxicity (30, 31).

In a phase 3 trial, maribavir at 100 mg BID did not prevent CMV disease (32). In another phase 3 trial, brincidofovir did not reduce CSCI at week 24 and was associated with significant gastrointestinal toxicity (33). Two systematic reviews focused on the effects of antiviral prophylaxis in allo-SCT recipients (11, 12). In both analyses, none of the drugs previously described showed reduction in all-cause mortality. Moreover, IV immunoglobulins or CMV-specific immunoglobulins are not recommended for prophylaxis of CSCI (34).

In 2017, the FDA approved LET to prevent CSCI in adult allo-SCT recipients (35). In the registration trial, prophylaxis with LET was started a median of 9 days after allo-SCT and administered through week 14. It was significantly associated with lower all-cause mortality than placebo through week 24 after allo-SCT (15). Patients considered at high risk of CSCI benefitted the most from antiviral prophylaxis. However, multiple CYP3A- and OATP1B1/3-mediated drug interactions may occur, especially when LET is co-administered with cyclosporine. Interestingly, LET does not appear to have significant hematologic or extra-hematologic toxicity.

Prospective randomized clinical trials are the statistical “gold standard” to evaluate the safety and efficacy of novel therapeutic agents. However, inclusion criteria are often very stringent, and the reproducibility of their results in real-world practice remains to be confirmed. Real-world studies have become increasingly important in their role of providing evidence of safety and efficacy in larger and more representative patient populations (36). Overall, they provide physicians with important clinical findings outside the context of clinical trials. Moreover, more rigorous methodology has greatly enhanced their quality, to the point that regulatory agencies such as the FDA and EMA currently recognize their potential value (37). Agencies underline the importance of real-life research in assessing marketed products and their life cycle, including development/monitoring and regulatory decision-making.

Within the setting of CMV prophylaxis, transplant programs will have to determine whether prophylaxis with LET is associated with survival benefits that offset the risk of toxicity and justify costs. In the present study, we described the most extensive real-world experience to date of prophylaxis with LET in allo-SCT patients, highlighting the reproducibility of the safety and efficacy of this commercially available antiviral agent. Although a stringent comparison of our findings with the registration trial is not possible, it is of note that only 40% of the enrolled patients experienced low to mild AEs that were easily managed. In our real-world experience, the efficacy of prophylaxis with LET was confirmed. Despite differences from the registration study in terms of baseline patient characteristics, none were significantly associated with treatment discontinuation or CSCI. Of note, the cumulative incidence of CSCI did not differ between our study and that of the registration trial in both observation periods, *i.e.* 5.4% versus 7.7% within day +100, and 18.1% versus 17.5% within day +168, respectively. In particular, we did not observe an increased frequency of CSCI in patients considered at high risk of this event at baseline versus those at low risk, suggesting that prophylaxis abrogated the impact of this variable on the study endpoint. Of note, the cumulative incidence of mortality at day +100 and at day +168 was higher than that reported in the registration trial (6.4 versus 1.5% and 7.4 versus 1.8%, respectively). This was probably due to differences in baseline prognostic characteristics and/or inclusion criteria between our study and the registration trial.

Other real-world experiences have also been published. An Italian study compared 45 patients undergoing prophylaxis with LET with a retrospective cohort that did not receive prophylaxis (38). Results showed that prophylaxis was highly effective and safe in reducing the incidence of CSCI when administered from day 0 to day +100. The incidence of CSCI at day +100 was significantly lower in patients who received prophylaxis than in those who did not (8% versus 44%, respectively). In another retrospective real-world study on 80 patients, prophylaxis was started after neutrophil engraftment, around the third week after allo-SCT (39). The incidence of CSCI at day +100 was 14%, lower than in the retrospective cohort not administered LET (41%).

The strength of the present study are the sample size, that is probably the largest one published so far, assessing the real word experience of LET prophylaxis, and the multicenter characteristic, comprising 17 centers in Italy. Nevertheless, this study has some limitations: first, the absence of a control group to make a comparison led to difficult interpretation of results; second, the results confirm what has been reported in other real-life studies, without adding new clinical information; and lastly, today we know that the presence of circulating infectious CMV particles is determined by virus isolation and degradation of free-floating viral DNA. For this reason, during LET prophylaxis the clinical relevance of CMV DNAemia should be critically considered, since the presence of DNAemia during letermovir prophylaxis may not represent a real CMV reactivation, but just an abortive infection (40). We don’t have this data in the few patients with DNAemia within day 100

There are still several unanswered questions on prophylaxis with LET, including potential benefits of its extension beyond day +100. Our real-life study showed that, after discontinuation of prophylaxis at day +101, some 13% of patients experienced CMV reactivation, supporting the prolongation of its administration. Moreover, a notable increase in reactivation was observed after week +19. An ongoing phase 3 clinical trial is currently evaluating the safety and efficacy of prophylaxis with LET beyond day +100 (41), focusing on the hypothesis that prolonged prophylaxis until day +200 is superior to placebo in preventing long-term CMV reactivation.

Moreover, the use of LET can reduce NRM. In a posthoc analysis performed to investigate the effects of LET on all-cause mortality, the incidence of all-cause mortality in the LET group was similar in patients with or without clinically significant CMV infection (42). In contrast, in the placebo group, all-cause mortality was higher in patients with versus those without clinically significant CMV infection, despite the use of pre-emptive therapy for CMV infection. These results suggest that there may be a benefit to avoiding clinically significant CMV infection and potentially toxic antivirals such as ganciclovir.

There are currently scanty data on the cost-effectiveness of prophylaxis with LET (43). However, in a recent study, prophylaxis in adult patients compared with no-prophylaxis showed favourable cost-effectiveness for the Italian National Health Service (44).

In conclusion, our real-world analysis reports similar efficacy findings to those of the registration trial. However, the costs of CMV prophylaxis may be prohibitive in countries with socioeconomic healthcare issues.

## Data Availability Statement

The raw data supporting the conclusions of this article will be made available by the authors, without undue reservation.

## Ethics Statement 

The studies involving human participants were reviewed and approved by Ethics Committee of Grande Ospedale Metropolitano “Bianchi-Melacrino-Morelli” of Reggio Calabria, Italy. The patients/participants provided their written informed consent to participate in this study.

## Author Contributions 

Study concepts: MMa, AMC, and CC. Quality control of data and algorithms: MMa, APit, GP, GT, MG, and CC. Data analysis and interpretation: MMa, APit, GP, GT, MG, and CC. Statistical analysis: APit, GT, and MG. Manuscript preparation: MMa, APit, GP, GT, MG, and CC. Manuscript editing: MMa, BB, and CC. Critical revision of the article: MMa, APit, BB, GP, GT, MG, CC, GMa and GMu. Data collection: AC, VF, APic, ST, CI, PC, DP, GMu, APa, AV, BS, GS, NM, SL, MC, LP, BL, AF, FC, EM, LG, AB, MMu, RS, NR, SM, PMa, PMu, IA, CS, FAC, and MP. All authors contributed to the article and approved the submitted version.

## Conflict of Interest

The authors declare that the research was conducted in the absence of any commercial or financial relationship that could be construed as a potential conflict of interest.

## Publisher’s Note

All claims expressed in this article are solely those of the authors and do not necessarily represent those of their affiliated organizations, or those of the publisher, the editors and the reviewers. Any product that may be evaluated in this article, or claim that may be made by its manufacturer, is not guaranteed or endorsed by the publisher.

## Abbreviations

AE, adverse event; allo-SCT, allogeneic stem cell transplant; AML, acute myeloid leukaemia; CMV, cytomegalovirus; CSCI, clinically significant CMV infection; GVHD, graft-versus-host disease; HLA, human leukocyte antigen; LET, letermovir; MRD, matched related; MUD, matched unrelated donor; NRM, non-relapse mortality.
